# Clinical and cost effectiveness of a psychosocial goal‐setting and manualised support intervention (NIDUS‐Family) for independence in dementia at home

**DOI:** 10.1002/alz70858_097772

**Published:** 2025-12-24

**Authors:** Jessica Budgett

**Affiliations:** ^1^ Queen Mary University of London, London, London, United Kingdom

## Abstract

**Background:**

Dementia care guidelines recommend personalised post‐diagnostic support, but few receive it. NIDUS (New Interventions for Independence in Dementia)‐Family intervention is fully manualised and designed to be delivered by trained, supervised, non‐clinical facilitators using accessible digital online modules and resources. We investigated the clinical and cost‐effectiveness of home‐based goal setting plus NIDUS‐Family in supporting people with dementia and their carers achieve personalised goals.

**Method:**

This two‐arm, single‐masked, multi‐site, randomised trial recruited patient–carer dyads from community settings. Based on their personalised goals, dyads selected modules involving behavioural interventions, carer support, psychoeducation, coping skills, enablement, and environmental adaptations. The intervention involved 6‐8 video‐call, telephone, or in‐person sessions over 6 months. The primary outcome was goal attainment scaling (GAS) score at 12 months. Cost‐effectiveness was evaluated using Quality Adjusted Life Year (QALY) from health and personal social services and societal perspectives, at £20,000‐£30,000 decision thresholds for QALY gained, compared to usual care over 12 months. Analyses were intention‐to‐treat. Trial registration: ISRCTN11425138

**Result:**

Of 302 eligible dyads, 204(67·5%) were randomised to intervention and 98 (32·4%) to control. 247(82%) dyads completed the primary outcome, which favoured the intervention (mean GAS score at 12 months 58·7 [SD 13·0; *n* = 163] *vs* 49·0 [14·1; *n* = 84]; adjusted difference in means 10·23 [95% CI 5·75–14·71]; *p* <0·001). 178 (58.9%) dyads provided cost data at 12 months. From health and personal social services perspectives, there was 91% probability that NIDUS‐Family was cost‐effective compared to usual care. There was 89% and 87% probability that NIDUS‐Family was cost‐effective compared to usual care from personal social services and societal perspectives respectively. Intervention participants accrued on average £8934 (37%) less costs than control participants (95% CI ‐£59,460 to £41,592).

**Conclusion:**

NIDUS‐Family is a clinically and cost‐effective personalised care intervention that delivers tailored facilitated support alongside online digital resources for people with dementia and family carers. Utilising scalable individualised goals as both an outcome measure and to shape the support means that this intervention holds significant potential for widely scalable implementation across diverse settings. Given its benefits, NIDUS‐Family should be part of routine dementia care and an implementation study is in progress.

